# Using a Live Analysis System to Study Amyloplast Replication in *Arabidopsis* Ovule Integuments

**DOI:** 10.21769/BioProtoc.5333

**Published:** 2025-06-05

**Authors:** Makoto T. Fujiwara, Rin Arakawa, Tomoko Abe, Ryuuichi D. Itoh

**Affiliations:** 1 Department of Materials and Life Sciences, Faculty of Science and Technology, Sophia University, Tokyo, Japan; 2 Nishina Center, RIKEN, Wako, Saitama, Japan; 3 Department of Chemistry, Biology and Marine Science, Faculty of Science, University of the Ryukyus, Senbaru, Nishihara, Okinawa, Japan

**Keywords:** Amyloplast, Conventional fluorescence microscope, Live cell imaging, Proplastid, Stromule

## Abstract

Amyloplasts, non-photosynthetic plastids specialized for starch synthesis and storage, proliferate in storage tissue cells of plants. To date, studies of amyloplast replication in roots and the ovule nucelli from various plant species have been performed using electron and fluorescence microscopy. However, a complete understanding of amyloplast replication remains unclear due to the absence of experimental systems capable of tracking their morphology and behavior in living cells. Recently, we demonstrated that *Arabidopsis* ovule integument could provide a platform for live-cell imaging of amyloplast replication. This system enables precise analysis of amyloplast number and shape, including the behavior of stroma-filled tubules (stromules), during proplastid-to-amyloplast development in post-mitotic cells. Here, we provide technical guidelines for observing and quantifying amyloplasts using conventional fluorescence microscopy in wild-type and several plastid-division mutants of *Arabidopsis*.

Key features

• Novel approach for investigating amyloplast differentiation and replication in plant cells.

• Detection of stroma-labeled amyloplasts in whole-mount ovules using conventional fluorescence microscopy.

• Facilitates quantitative and comparative analysis of amyloplast proliferation using various *Arabidopsis* resources.

• Enables high-resolution analysis of changing amyloplast and stromule morphologies in living cells.

## Graphical overview


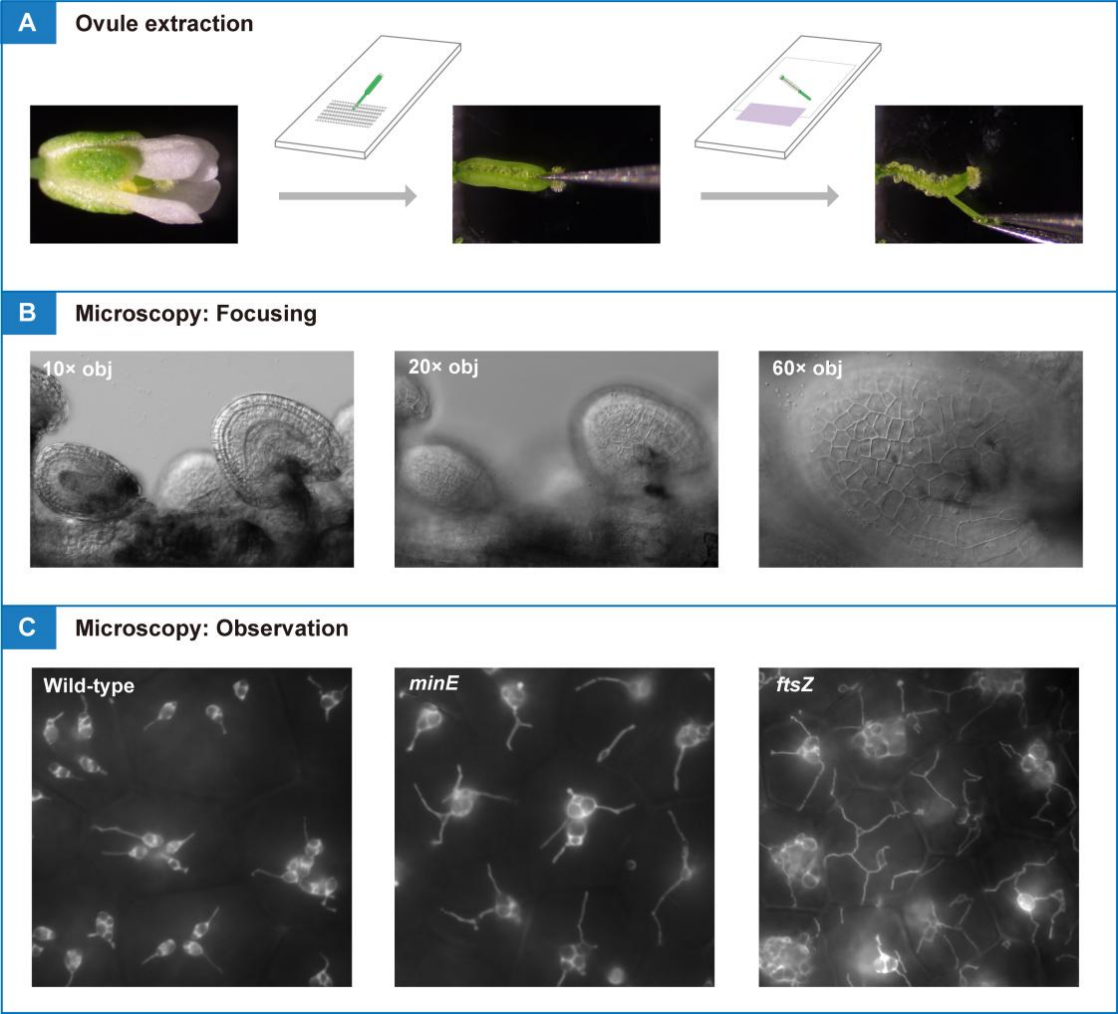


**Pistils or siliques are dissected and subjected to conventional fluorescence microscopy for amyloplast observation and quantification in living tissue**

## Background

Plastids are double membrane–bound organelles found in the cells of seed plants that exhibit unique features of functional and morphological differentiation. These include chloroplasts in leaves, chromoplasts in flowers and fruits, and leucoplasts in general non-photosynthetic tissues [1]. Amyloplasts are non-photosynthetic plastids specialized for starch synthesis and storage. Amyloplasts develop from undifferentiated proplastids in meristematic tissues and are distributed in storage tissues such as roots, stems, and seeds, as well as in gravity-sensing tissues (for example, the stem endodermis and root cap columella) [2]. As starch is an important source of nutrients in human diets, the differentiation and proliferation of amyloplasts have long been an important area of crop biology research.

Plastids possess their DNA and typically replicate by binary fission [3,4]. In leaf mesophyll cells of the model plant *Arabidopsis thaliana*, chloroplast division is initiated by the formation of the stromal FtsZ ring and involves approximately 20 nuclear-encoded gene products located in the stroma, inner-envelope membrane, outer-envelope membrane, or cytoplasm [5,6]. In contrast, the mechanisms governing amyloplast proliferation remain largely unknown, partly due to the lack of an experimental system to track their number and behavior in specific cells.

We recently proposed that the *Arabidopsis* ovule integument could provide a platform for live-cell imaging of amyloplast replication and morphology, including the dynamics of stroma-filled tubules (stromules) [7]. This method may open a way to analyzing the function and regulation of plastid-division proteins during amyloplast proliferation in non-photosynthetic cells. In this protocol, we provide technical tips for conventional fluorescence microscopy of integument amyloplasts using wild-type (WT) and plastid-division mutants of *Arabidopsis*.

## Materials and reagents

**Biological materials**

**Figure 1.**
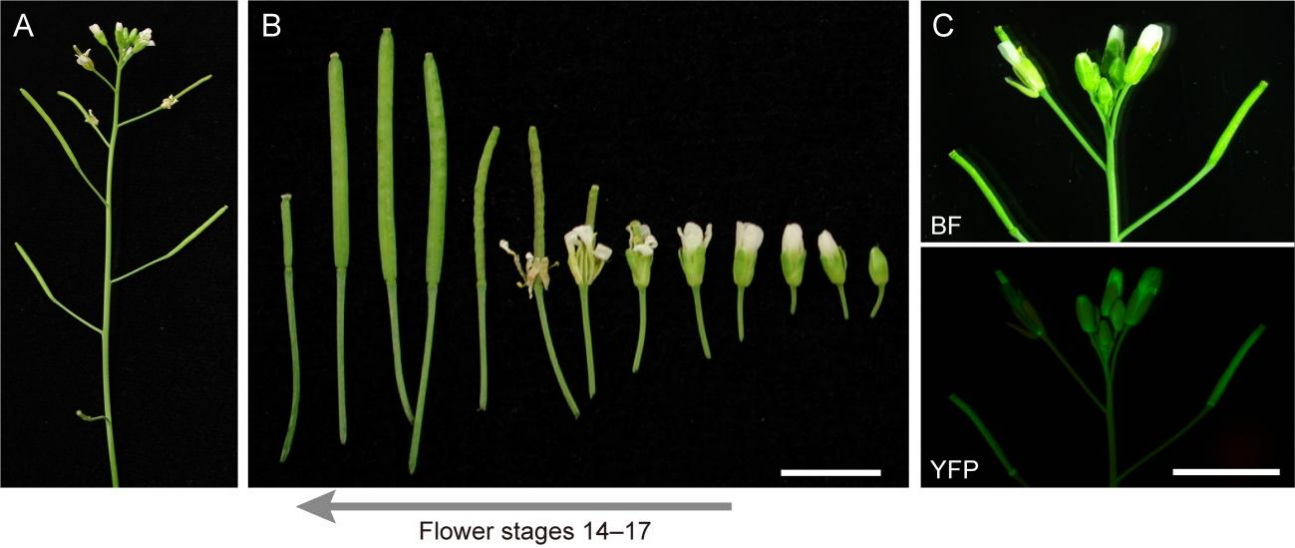
Reproductive stages of *Arabidopsis* suitable for analysis of amyloplast replication. (A) Primary inflorescence. (B) Flower stages 14–17 [11] used for ovule extraction. (C) Expression pattern of CaMV*35S* promoter-driven stroma-targeted YFP in a transgenic *Arabidopsis* line. Images of brightfield (BF) and yellow fluorescent protein (YFP) signals are shown. Scale bar, 5 mm.

1. Seeds of transgenic *Arabidopsis* lines with stable expression of stroma-targeted fluorescent proteins (see General note 1). This protocol presents data from transgenic lines in the Col background [8] and *minE* [9], and *ftsZ* triple [10] (hereafter, *ftsZ*) mutants, in which a transit peptide (the N-terminal 90 aa of AtFtsZ1-1 [8])-fused YFP is driven by the constitutive CaMV*35S* promoter (see Figure 1)

**Reagents**

1. Murashige and Skoog plant salt mixture (Wako Pure Chemical Industries, catalog number: 392-00591)

2. Sucrose (Wako Pure Chemical Industries, catalog number: 196-00015)

3. MES (Nacalai Tesque, catalog number: 21623-26)

4. Phyto agar (Duchefa Biochemie, catalog number: P1003.1000)

5. Jiffy-7 peat pellets, 44 × 42 mm (Jiffy International)

**Laboratory supplies**

1. Slide glass (Matsunami Glass, catalog number: S-2215)

2. Cover glass, 24 × 32 No. 1, thickness 0.13–0.17 mm (Matsunami Glass, catalog number: C024321)

3. Micropore surgical tape (3M, catalog number: 1530SP-0)

4. Paper tape (As One, catalog number: 6-691-01)

## Equipment

1. Stereomicroscope with 0.63× objective lens (Leica Microsystems, model: MZ10 F)

2. CCD camera (Olympus, model: DP26) with 0.63× adaptor, attached to Leica MZ10 F

3. Inverted fluorescence microscope with 10×, 20×, and 60× objective lenses (Olympus, model: IX71)

4. CMOS camera (Hamamatsu Photonics, model: ORCA-flash2.8) with a 1.0× adaptor, attached to Olympus IX71

5. Tweezers 1 (Fontax, No. 5 Taxal)

6. Tweezers 2 (Dumont, No. DU-55 Dumoxel)

## Software and datasets

1. Imaging software CellSens (Olympus, controller for DP26 camera)

2. Imaging software HCImage (Hamamatsu Photonics, controller for ORCA-flash2.8 camera)

## Procedure

**A. Plant cultivation and extraction of ovules for microscopy**

**Figure 2.**
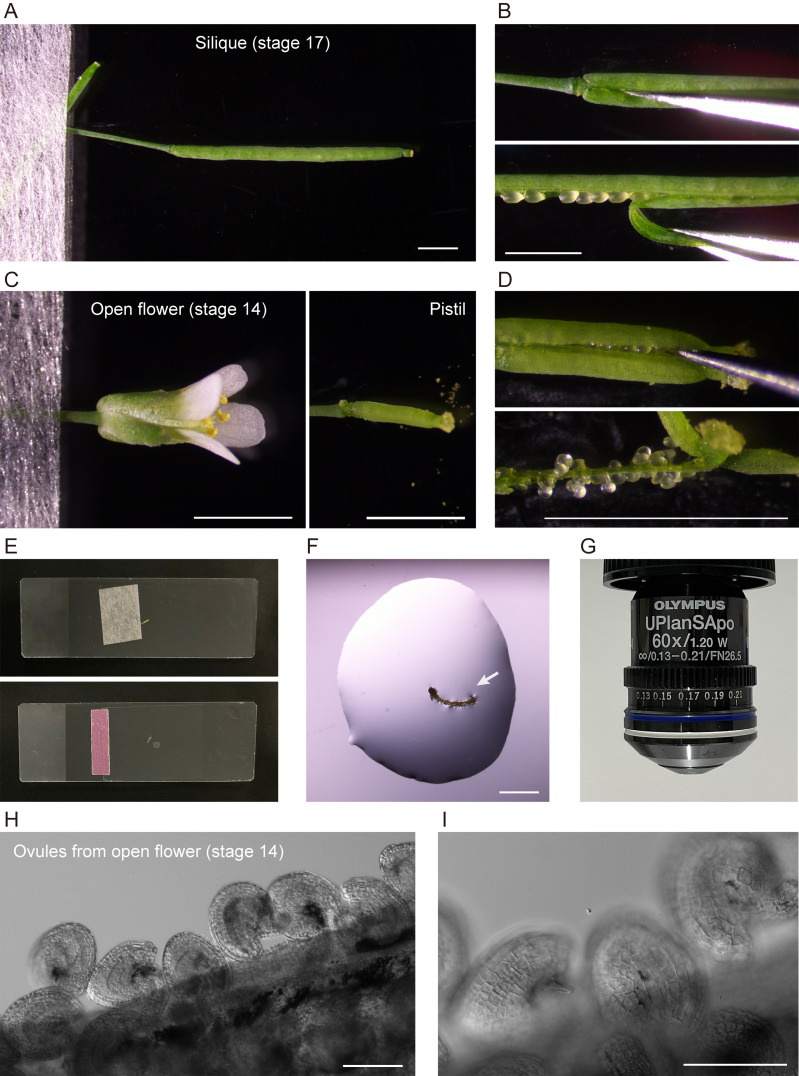
Extraction of ovules for microscopic analysis. (A,B) Sampling and dissection of a silique. (C,D) Sampling and dissection of a pistil from an open flower. (E) Slides used for dissection (top) and microscopy (bottom). (F) An extracted septum–ovule association (arrow) mounted in water. (G) 60× objective used for microscopy. (H, I) Ovules observed under 10× (H) and 20× (I) objectives. Scale bars, 2 mm (A–D, F) and 100 μm (H, I).

1. Sow seeds on Murashige and Skoog medium (pH 5.7) supplemented with 0.1% MES, 2% sucrose, and 0.7% agar (see General note 2). Treat the seeds in the dark at 4 °C for several days [9]. Germination occurs in one or several days after irradiation at 23 °C. After germination, transfer seedlings to Jiffy-7 peat pellets pre-soaked in water and grow under a 16/8 light/dark cycle of white light illumination at 23 °C for 5–6 weeks (see General note 2; Figure 1).

2. Prepare two glass slides. One (slide 1) is used for ovule extraction. The other (slide 2), together with 1–3 layers of colored tape as a spacer, is used for microscopy analysis (see General note 3).

3. Excise an intact flower or silique from an inflorescence stem of an adult plant (see General note 4).

4. Place the flower or silique (segment) on slide 1. Fix the organ in place using surgical tape by securing the pedicle at one point so that it does not move during dissection (Figure 2A–E).

5. To extract ovules from a silique at flower stages 16–17, remove sepals, petals, and stamens from the silique, if attached, using basic tweezers (tweezers 1) under a stereomicroscope at zoom 2.0–3.2×. Peel off either or both valves (this is optional) from the silique (Figure 2A, B).

6. To extract ovules from a pistil at flower stages 13–16, remove sepals, petals, and stamens from an open flower using extra-fine tweezers (tweezers 2) under a stereomicroscope at zoom 6.3×. Peel off either or both valves (this is optional) from the pistil (Figure 2C, D).

7. Apply 40–70 µL of deionized water or liquid Murashige and Skoog medium onto slide 2. Transfer a septum–ovule association obtained by dissection to the water drop (Figure 2F).

8. Mount a coverslip carefully onto the slide (see General note 5). Gently apply the mounting medium between the coverslip and the slide, so that it just fills the space between them (Figure 2E).

9. Confirm the extraction of intact ovules by low-magnification light microscopy (Figure 2H, I).

10. Observe amyloplasts in outer ovule integument (epidermal or oi2 [12]) cells by high-magnification fluorescence microscopy using a 60×/1.20 NA water-immersion objective (Figure 2G; see section B).

**B. Observation of amyloplasts by conventional fluorescence microscopy**

**Figure 3.**
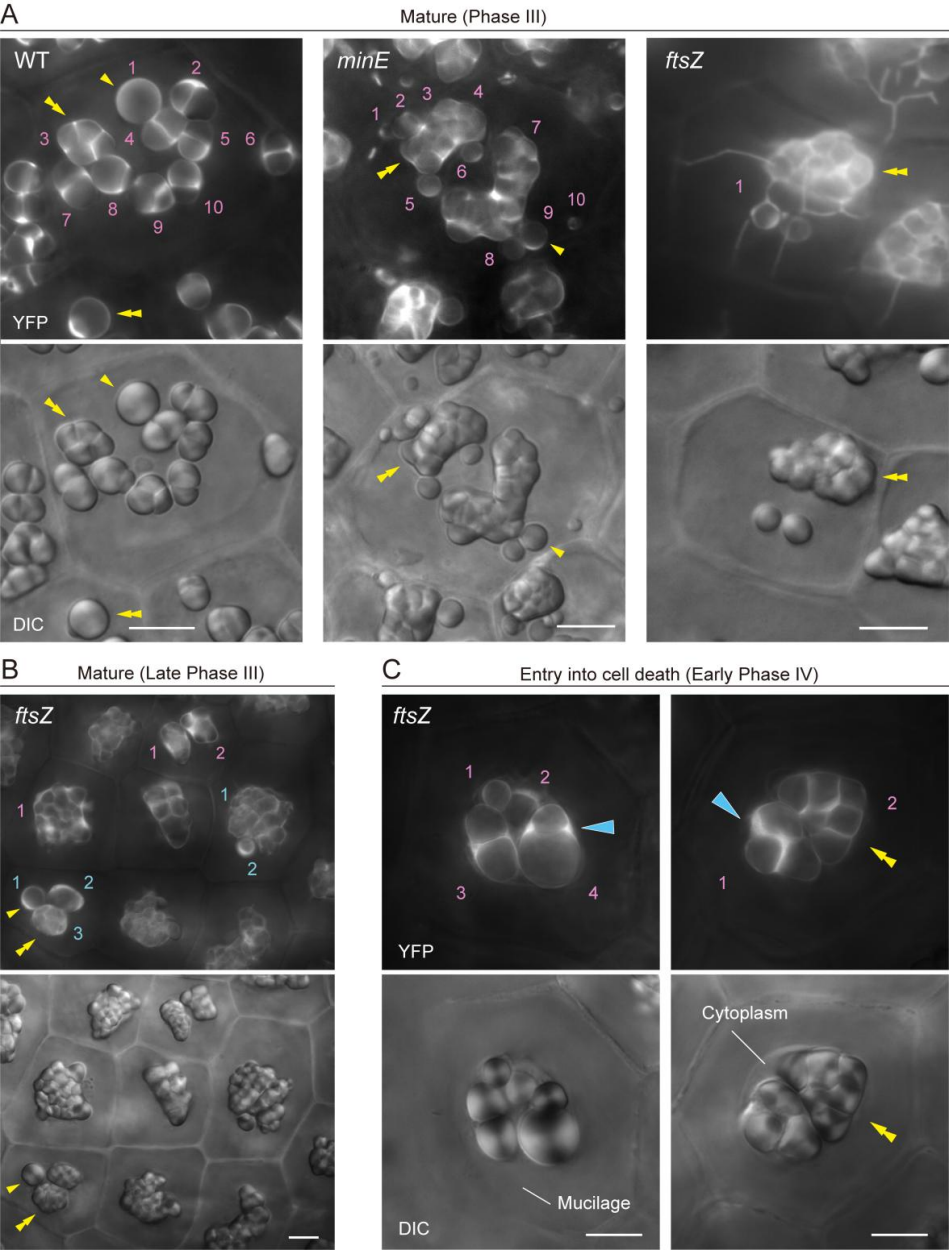
Amyloplast count and morphology in integument cells of wildtype (WT), *minE*, and *ftsZ* plants. (A) Integument cells of WT, *minE*, and *ftsZ* plants with developed amyloplasts (Phase III). (B) Integument cells of *ftsZ* with fully mature amyloplasts (late Phase III). (C) Integument cells of *ftsZ* upon entry into the cell death stage (early Phase IV). (A–C) show images of stroma-targeted YFP (top) and DIC (bottom). Single and double yellow arrowheads indicate single and compound starch grains, respectively. Cyan single arrowheads indicate the accumulation of stromal YFP in amyloplasts. Numbers indicate amyloplast counts per cell. Scale bars, 10 µm.

**Figure 4.**
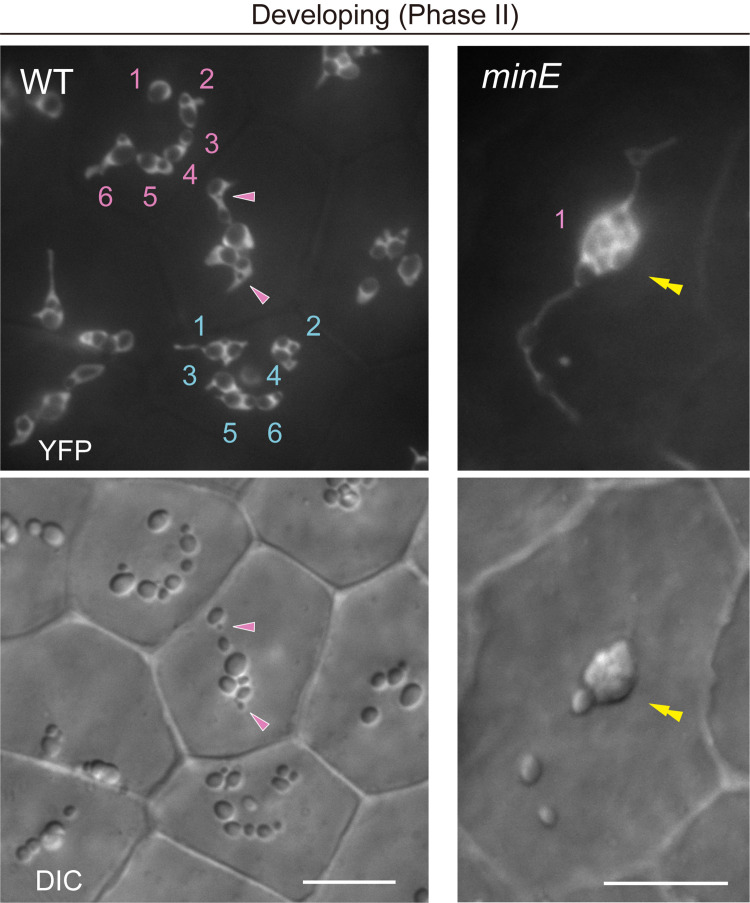
Count and morphology of middle-developed amyloplasts in wildtype (WT) and *minE* integument cells. Images show stroma-targeted yellow fluorescent protein (YFP) and differential interference contrast (DIC). Single and double arrowheads indicate the position of stroma-accumulated YFP signals and compound starch grains, respectively. Numbers indicate amyloplast counts per cell. Scale bars, 10 μm.

**Figure 5.**
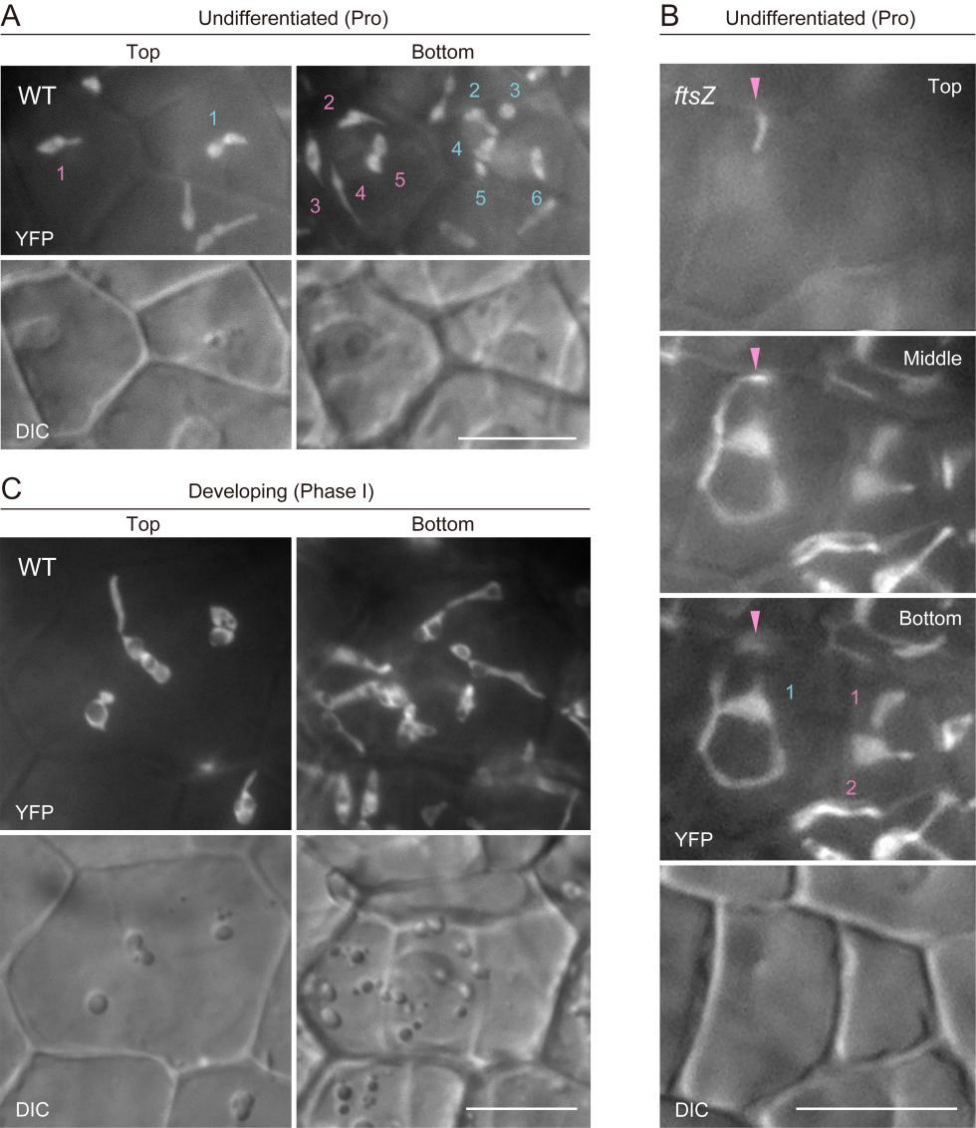
Count and morphology of proplastids and early differentiating (Phase I) amyloplasts in integument cells of wild-type and *ftsZ* plants. (A–C) Images of stroma-targeted yellow fluorescent protein (YFP) and differential interference contrast (DIC). Single arrowheads indicate the location of an extending stromule along the z-axis. Numbers indicate amyloplast counts per cell. Scale bars, 10 μm.

1. Turn on the halogen lamp, mercury lamp, camera controller (computer), and digital camera.

2. Place slide 2 on the stage of the inverted fluorescence microscope.

3. To perform counting and morphological characterization of amyloplasts, use a 60× water-immersion objective (Figure 2G). Microscope settings for the detection of YFP are described in Table 1.

4. Start the camera controller software HCImage (Hamamatsu Photonics). The settings of the mercury lamp and camera for the detection of YFP fluorescence are described in Table 2.

5. Explore and focus on individual ovules at 60× through eyepieces using differential interference contrast (DIC) optics.

6. Perform image acquisition (Figures 3–5). For time-lapse fluorescence microscopy, we manually focus on individual cells and capture images for up to 60 min. Each image is captured every 30 s or 1 min.

**Table 1.** Settings for standard epifluorescence microscopy

Item	Products/values (maker)
Objective	10× dry (N.A. 0.40), 20× dry (N.A. 0.75) 60× water-immersion lens (N.A. 1.20)
Light source for DIC observation	Halogen lamp at 10%–50% output (TH4-100, Olympus)
Light source for fluorescence observation	Mercury lamp at 3%–12% output (U-HGLGPS, Olympus) Plus ND filter 25
Optical filters for YFP	BP490-500HQ (ex) (Olympus) FF506-DiO3 (di) (Semrock) FF01-545/55 (em) (Semrock)

**Table 2.** Settings of the camera and software for fluorescence detection

Item	Products/values
Image	12 bit, 1920 × 1440 pixel
Binning	1
HCImage software for single image capture: Gain (range: 0–255) Exposure	100 500 ms
HCImage software for time-lapse microscopy: Gain (range: 0–255) Exposure	150 500 ms

## Data analysis

**A. Morphological characterization and counting of mature amyloplasts in WT**

1. Single and compound starch granules in amyloplasts can be distinguished using both brightfield (DIC) and YFP fluorescence imaging (Figure 3A, WT). Stromal YFP does not enter the interior of starch grains but accumulates in a small space(s) between grains.

2. The amyloplast distribution from the top to the bottom of a tile-formed cell is checked using both DIC and stromal YFP, or by DIC imaging alone. The amyloplast number per cell is obtained with relative ease.

**B. Morphological characterization and counting of mature amyloplasts in plastid-division mutants**

1. The observation points essentially resemble those of WT amyloplasts, but the following observations are specific to plastid-division mutants. Integument cells of chloroplast division-inhibition mutants often contain YFP-labeled and starchless plastids. Such starchless plastids occur occasionally in WT but frequently appear in severe plastid-division inhibition mutants.

2. In general, an unusually elevated number of starch granules occurs inside giant amyloplasts.

3. Another structural factor in amyloplasts of plastid-division mutants is the activation of stromule formation in cells. Starch grains are often detected within stromules. In particular, *ftsZ* mutants tend to show extended stromule formation until late Phase III or early Phase IV amyloplasts. In such cases, a stromule-starch grain(s)-giant amyloplast association should be counted as a single amyloplast (Figure 3A, *minE* and *ftsZ*).

4. In *ftsZ* mutants, activated stromules disappear in late Phase III or early Phase IV (Figure 3B, C). Although amyloplast counting is more difficult at early Phase IV than at late Phase III, because of limited cytoplasmic space, the final amyloplast number per cell is revealed.

**C. Morphological characterization and counting of amyloplasts during middle development in WT and plastid-division mutants**

1. During the middle development of amyloplasts, the number as well as the starch grain morphology in each amyloplast from both WT and plastid-division mutants should be judged using both DIC and stromal YFP images. Amyloplast counting is difficult when using only DIC images (Figure 4, WT). The distribution patterns of stromal fluorescent proteins, the position and shape of starch grains, and their temporal change during the observation enable these characterizations.

2. If starch grain-containing stromules are not separated from the main body of amyloplasts, they are regarded as a single amyloplast (Figure 4, *minE*).

**D. Morphological characterization and counting of proplastids or early differentiating amyloplasts in WT and plastid-division mutants**

1. To assess amyloplast proliferation phenotypes during ovule development in WT and plastid-division mutants, information on the proplastid number per cell is critical. The following four factors are essential for this analysis:

- A high level of stromal fluorescence signal for proplastid labeling.
- Clear-cut fluorescence detection conditions to capture the proplastid configuration.
- Counting by live observation, rather than by using static fluorescence images only.
- Counts that recognize the entire proplastid configuration within a given cell by shifting the focus from the top to the bottom of the cell.
  If one or more of the above factors are unsatisfactory, precise counting of proplastids may become difficult.

2. Selected captured images of proplastids in integument cells of WT (Figure 5A) and *ftsZ* (Figure 5B) are presented as examples. In WT, proplastids are often distributed at the top and bottom surfaces of integument cells and assume a dumbbell shape. In *ftsZ*, proplastids extend stromules in three dimensions. The extent of stromule elongation and branching is relatively modest in young cells compared with that in more mature and expanded cells. The small volumes of integument cells in mature ovules, prior to their development after fertilization [13,14], enable proplastid counting in *ftsZ*.

3. Counting and analysis of early differentiating (Phase I) amyloplasts are most difficult, as indicated in Figure 5C. Integument cells begin to expand, and differentiating amyloplasts also grow larger. In addition, individual amyloplasts are often hard to distinguish.

## Validation of protocol

This protocol, or parts of it, has been used and validated in the following research article:

- Fujiwara et al. [7]. Principles of amyloplast replication in the ovule integuments of *Arabidopsis thaliana. Plant Physiology* (Figure 1F–K, M–O; Figure 2A–D; Figure 4A–D; Figure 5A–E; and Supplementary figures).

## General notes and troubleshooting

**General notes**

1. This system is compatible with commonly used fluorescent proteins, including GFP, CFP, YFP, and RFP.

2. Sowing seeds on 1%–3% sucrose-containing Murashige and Skoog media or directly into Jiffy-7 resulted in similar amyloplast development and proliferation results. We cultivated one plant per Jiffy-7. We do not recommend using >7-week-old plants for analysis.

3. Flower stages 13–14 [11] allow for the analysis of proplastids in integument cells. Flower stages 15–17 are suitable for examining developing or mature amyloplasts.

4. Selection of inflorescence stems: We primarily use primary to third inflorescence stems for analysis.

5. Take special care when extracting ovules and mounting samples and coverslips.
